# G-quadruplex Stabilization Fuels the ALT Pathway in ALT-positive Osteosarcoma Cells

**DOI:** 10.3390/genes11030304

**Published:** 2020-03-13

**Authors:** Roberta Amato, Martina Valenzuela, Francesco Berardinelli, Erica Salvati, Carmen Maresca, Stefano Leone, Antonio Antoccia, Antonella Sgura

**Affiliations:** 1 Department of Science, Roma Tre University, I-00146 Rome, Italy; Roberta.amato@uniroma3.it (R.A.); martina.valenzuela@uniroma3.it (M.V.); Stefano.leone@uniroma3.it (S.L.); Antonio.antoccia@uniroma3.it (A.A.); antonella.sgura@uniroma3.it (A.S.); 2 BPM-CNR Institute of Molecular Biology and Pathology, National Research Council, 00185 Rome, Italy; erica.salvati@uniroma1.it; 3 Oncogenomic and Epigenetic Unit, IRCCS Regina Elena National Cancer Institute, 00144 Rome, Italy; carmen.maresca@ifo.gov.it

**Keywords:** telomeres, ALT, G-quadruplex, RHPS4, replicative stress

## Abstract

Most human tumors maintain telomere lengths by telomerase, whereas a portion of them (10–15%) uses a mechanism named alternative lengthening of telomeres (ALT). The telomeric G-quadruplex (G4) ligand RHPS4 is known for its potent antiproliferative effect, as shown in telomerase-positive cancer models. Moreover, RHPS4 is also able to reduce cell proliferation in ALT cells, although the influence of G4 stabilization on the ALT mechanism has so far been poorly investigated. Here we show that sensitivity to RHPS4 is comparable in ALT-positive (U2OS; SAOS-2) and telomerase-positive (HOS) osteosarcoma cell lines, unlinking the telomere maintenance mechanism and RHPS4 responsiveness. To investigate the impact of G4 stabilization on ALT, the cardinal ALT hallmarks were analyzed. A significant induction of telomeric doublets, telomeric clusterized DNA damage, ALT-associated Promyelocytic Leukaemia-bodies (APBs), telomere sister chromatid exchanges (T-SCE) and c-circles was found exclusively in RHPS4-treated ALT cells. We surmise that RHPS4 affects ALT mechanisms through the induction of replicative stress that in turn is converted in DNA damage at telomeres, fueling recombination. In conclusion, our work indicates that RHPS4-induced telomeric DNA damage promotes overactivation of telomeric recombination in ALT cells, opening new questions on the therapeutic employment of G4 ligands in the treatment of ALT positive tumors.

## 1. Introduction

Telomeres are nucleoprotein structures that protect the ends of linear chromosomes [1,2]. In humans, telomeres are composed of 10–15 kb of tandem arrays of TTAGGG repeats [3,4] bound by specialized proteins [5] that cooperatively conceal chromosome ends, impeding their processing as DNA double-strand breaks (DSBs) and contributing to genome stability. In cells lacking a telomere maintenance mechanism (TMM), telomeric DNA encounters progressive erosion at each cell duplication, eventually leading to replicative senescence. In somatic primary cells, this represents a tumor-suppressive mechanism and, as a consequence, continuous dividing cells (e.g., cancer cells) need to avoid senescence by the maintenance of stable telomere lengths. Most human cancers maintain their telomeres by reactivating the reverse transcriptase telomerase [6]. However, a significant fraction (10–15%) utilizes a homologous recombination (HR) based TMM known as alternative lengthening of telomeres (ALT) [7,8]. ALT is frequently found in osteosarcomas, soft tissue sarcomas [9], pancreatic neuroendocrine tumors [10], glioblastoma multiforme, oligodendrogliomas, medulloblastomas [11], and neuroblastomas [12]. High throughput genome sequencing of cells and tumors displaying the ALT phenotype has identified mutations in the ATRX/DAXX/H3.3 complex. ATRX is a chromatin remodeling factor of the Snf2 family [13], which, cooperating with the histone chaperone DAXX, enables the incorporation of the histone variant H3.3 into telomeric [14,15] and pericentromeric chromatin [16]. Two distinct mechanisms mediated the ALT pathway: the first one is a RAD51-dependent recombination mediated process (mechanistically similar to the yeast Type I ALT) [17,18], whereas the second one is RAD51-independent and is probably mediated by break-induced replication (BIR) (RAD52- and POLD3-dependent and mechanistically similar to the yeast Type II ALT) [19,20]. Due to their specific presence in cancer cells, and minimal or absent expression in most somatic cells, TMMs are specific cancer biomarkers. In particular, telomerase is a very appealing target for the development of broad-spectrum antineoplastic therapeutics and, in the last 20 years, several strategies have been proposed in order to inhibit telomerase activity [21]. On the other hand, ALT cells do not display an exclusive enzymatic activity, rendering the development of specific ALT inhibitors very puzzling [22]. Nonetheless, several papers report that telomere-targeting agents such as G-quadruplex (G4) ligands are able to inhibit cell proliferation in both telomerase and ALT cancer cells [23,24,25,26]. This class of compounds binds and stabilizes non-canonical secondary structures that arise in G-rich regions of the genome, such as telomeres, gene promoters, and replication start sites. In this context, the pentacyclic acridine compound RHPS4 is one of the most effective telomeric G4-stabilizing molecules. In particular, RHPS4 determines telomere deprotection, leading to replication stress, telomeric fusions, anaphase bridges, cell proliferation blockage, and radiosensitization, as shown in large panel of cancer cells in vitro and in vivo [23,27,28,29,30,31,32,33]. RHPS4-mediated inhibition of cell proliferation was also observed in ALT-positive cell lines such as GM847DM SV40-immortalized human skin fibroblasts and SAOS-2 osteosarcoma in different experimental settings [24,34] and, in general, the efficacy of telomeric G4 ligands in ALT cells is associated to the interference of G4 structures with telomere recombination [35,36] even if no direct evidence is available in the literature. More recently, other authors have indicated that G4 stabilization may play a role in the ALT pathway, linking mutations in ATRX with reduced efficacy of G4 resolution and/or with augmented replicative stress and BIR at telomeres [37,38]. Therefore, in order to clarify whether telomeric G4 stabilization is able to hinder or to fuel the ALT pathway, the cardinal hallmarks of ALT were analyzed in three different osteosarcoma cell lines, two ALT-positive (U2OS and SAOS-2) and one ALT-negative (HOS) used as a control, treated with the G4 ligand RHPS4. Results indicated that RHPS4-mediated inhibition of cell proliferation was unlinked to ALT, as no suppression in any of the analyzed parameters was found. On the contrary, the frequency of telomeric doublets, telomeric damage, ALT-associated PML bodies (APBs), telomeric sister chromatid exchanges (T-SCEs) and c-circles was significantly increased upon RHPS4 treatment exclusively in ALT cells. Collected evidence suggests that G4-ligands may increase replication stress and DNA damage at telomeres, stimulating recombination mechanisms in ALT-positive cells.

## 2. Materials and Methods 

### 2.1. Cell Lines and Culture Conditions

Media, supplements, and plasticware for cell culture were purchased from Euroclone (Euroclone, Pero, MI, Italy). U2OS (p53 wt, MDM2 overexpressed and ATRX mutated) [39,40] and HOS (p53 mutated and ATRX wt) [40,41,42] were routinely maintained in Dulbecco’s Modified Eagle’s Medium High Glucose (4500 mg/l) supplemented with 10% FBS, 2 mM L-glutamine, sodium pyruvate with 100 units/mL penicillin and 100 µg/mL streptomycin. SAOS-2 (p53 mutated and ATRX mutated) [40,43] were maintained in Minimum Essential Medium with Earle’s Balanced Salt Solution (MEM/EBSS) with 10% FBS, 2 mM L-glutammine, 100 units/mL penicillin, and 100 µg/mL streptomycin. Telomerase activity (TA) was tested in all the cell lines by TRAP-assay, confirming that only HOS cells are telomerase-positive. All the aforementioned cell lines were maintained at 37 °C in a 5% CO2 and 95% air atmosphere. All cell lines were routinely mycoplasma tested.

### 2.2. Chemical Compound and Treatments

The pentacyclic acridine, 3,11-difluoro-6,8,13-trimethyl-8Hquino [4,3,2-kl] acridinium methosulfate (RHPS4; Tocris, Bristol, UK), was dissolved in dimethyl sulfoxide (DMSO). Cells were treated with the drug 6 h after plating and incubated for 120 h. The dishes used as control were treated only with DMSO. The drug was freshly prepared from frozen aliquots for each set of experiments. If not differently stated, all the experiments were performed treating cells with their respective IC_50_ calculated 120 h after treatment.

### 2.3. Growth Curves and cPDL Calculation

Cells were seeded at the density of 5000 cells/cm^2^ and treated using increasing concentrations of RHPS4. After 72, 96, and 120 h cells were detached and counted. Cumulative population doubling level (cPDL) was calculated as follows: cPDL = log 2(N_f_/N_0_), where N_f_ is the final cell number and N_0_ is the initial number of seeded cells. Results were obtained from three independent experiments.

### 2.4. Sulforhodamine B (SRB) Assay

Exponential growing cells were harvested, counted, and seeded in 96-well plates at different density for each line (HOS: 500 cells/well; U2OS: 1000 cells/well; SAOS: 4000 cells/well). Optimal seeding density was determined to ensure exponential growth during a 5-day assay (120 h). The SRB assay was performed as previously described [44], with minor modifications. Cell were fixed in 10% cold trichloroacetic acid, incubated at 4 °C for 1 h and then washed with deionized water. Cells were stained with 200 µl/well of 0.1% SRB (Sigma-Aldrich, Co., St. Louis, MO, USA) for 30 min and washed four times with 1% acetic acid. Plates were air-dried at room temperature (RT), and stained proteins were solubilized with 200 µl/well of 10 mM unbuffered Tris base (tris(hydroxymethyl) aminomethane) (Sigma-Aldrich Co., St. Louis, MO, USA). Optical density was read at 530 nm with a Victor plate-reader (VICTOR X3 Multilabel plate reader, PerkinElmer, Waltham, MA, USA). Experiments were repeated five times.

### 2.5. Flow Cytometric Analysis

Cells were treated with RHPS4 for 72, 96 and 120 h, pulsed with 10 µM bromodeoxyuridine (BrdU) in the last 30 min and then fixed and analyzed. Briefly, each sample was fixed, permeabilized, and the histones were dissociated with 2 M HCl as previously described [30]. BrdU positive cells were detected with an anti-BrdU primary antibody diluted 1:100 (DAKO Cytomatation, Glostrup, Denmark) and with an anti-mouse-Alexa488 conjugated diluted 1:100 (Invitrogen, Life Technologies, Carlsbad, CA, USA). Both antibodies were incubated for 1 h RT in the dark. All samples were counterstained with propidium iodide (PI) for DNA/BrdU biparametric analysis. BrdU positive cells were gated, and the relative percentage was calculated by Cytexpert software (Beckman Coulter, Inc., San Diego, CA, USA).

### 2.6. Collection of Chromosome Spreads

Chromosome spreads were obtained following 4 h incubation in 5 × 10^–6^ M colchicine (Sigma-Aldrich). Spreads of chromosomes were prepared following standard procedure consisting of treatment with a hypotonic solution (75 mM KCl) for 20 min at 37 °C, followed by fixation in freshly prepared Carnoy solution (3:1 *v/v* methanol/acetic acid). Cells were then dropped onto slides, air-dried and utilized for cytogenetic analysis.

### 2.7. Chromosome Orientation–FISH (CO–FISH) Analysis 

Cell lines subcultured in the presence of 5’-bromo-2’-deoxyuridine (BrdU, Sigma Aldrich) at a final concentration of 2.5 × 10^–5^ M and were then allowed to replicate their DNA once at 37 °C overnight (ON). Cells were then collected, and chromosome spreads were prepared as described above. CO–FISH was performed as previously described [45] using a (TTAGGG)_3_ probe labeled with Cy3 and a (CCCTAA)_3_ probe labeled with FITC (Panagene, Yuseong-gu, Korea). Images were captured with an Axio Imager.M1 equipped with a CCD camera. T-SCEs were scored only when the double signals were visible with both the Cy3 and FITC probes. Experiments were repeated three times and 4000 chromosome ends were analyzed for each line and condition. G-SCE was evaluated by scoring the number of chromosomes with normal (trans) and recombined (cis) CO–FISH signals configuration. To correct cis frequencies for multiple crossovers, we used the following expression [46]:

(1) $$\overset{\infty }{\underset{n=0}{\sum }}\left\{\left[\mu^{\left(2n+1\right)}e^{-\mu }\right]/\left[\left(n+1\right)!\right]\right\}$$

which is equivalent to

(2) μ=[−ln(1−2γ)]/2

in which the frequency of SCEs that would be necessary to account for the number of cis events represents the mean (μ) of a Poisson distribution, whose sum of odd-numbered terms equals the observed cis frequency (γ). The latter equation was used to convert the observed cis frequencies into estimates of the true crossover frequency, also referred to as the SCE equivalent frequency. For this purpose, 4000 chromosome ends were scored in three independent experiments. Moreover, double color telomere doublets, a marker of telomere fragility, were counted for each chromosome end considering both the following configuration: overlapping double green and red signals; red signal beside a green signal.

### 2.8. TIF Co-immunostaining

Cells were fixed with 4% paraformaldehyde (PFA) at 4 °C, permeabilized with 0.2% Triton X-100 at 4 °C and blocked in BSA 1% dissolved in phosphate-buffered saline (PBS) (*w/v*) at 37 °C. Samples were then co-immunostained overnight (ON) at 4 °C, using a rabbit polyclonal telomeric protein TRF1 antibody (Santa Cruz Biotechnology, Santa Cruz, CA, USA) in combination with a mouse monoclonal anti-53BP1 antibody (Millipore Corp. 290 Concord Rd, Billerica, MA, USA). After washes in PBS/BSA 1% samples were incubated with the secondary antibody (anti-mouse Alexa 546 and anti-rabbit Alexa 488, Invitrogen, Life Technologies, Carlsbad, CA, USA) for 1 h at 37 °C. Finally, slides were washed in PBS/BSA 1%, counterstained with DAPI (Sigma-Aldrich), and images were acquired with an Axio-Imager.M1 fluorescent microscope (Carl Zeiss, Jena, Germany) and analyzed using ISIS software (Metasystems, Milano, Italy). The frequency of DNA damage marker foci and TRF1/53BP1 colocalization dots per cell were scored in 100 nuclei in three independent experiments.

### 2.9. Immuno–FISH Experiments

Immunostaining with a rabbit polyclonal antibody against PML (E-11:sc-377390, Santa Cruz Biotechnology) or RPA32/RPA2 (phosphor-S33) (ab211877, Abcam, Cambridge, MA, USA) was performed as described above. After washing with 0.05% Triton X-100 in PBS for 5 min, cells were incubated with a secondary (goat) anti-rabbit antibody conjugated with Alexa 488 diluted in blocking solution for 1 h at room temperature (for PML or RPA2 detection). After immunostaining, telomeric FISH was performed as described in the previous paragraph. Images were taken using an Axio Imager.M1 (Carl Zeiss, Jena, Germany) equipped with a CCD camera and then analyzed using ISIS software (Metasystems, Milano, Italy). At least 100 nuclei in three independent experiments were analyzed to identify colocalization events.

### 2.10. Telomeric RNA FISH

Telomeric RNA FISH was performed as previously described [47,48], with minor modifications. Cells grown on glass microscope slides were incubated in ice-cold cytobuffer (100 mM NaCl, 300 mM sucrose, 3 mM MgCl2, 10 mM pipes, pH 6.8) for 30 s, washed in cytobuffer with 0.5% Triton X-100 for 30 s, and then fixed for 10 min in ice-cold 4% paraformaldehyde in PBS. Cells were dehydrated through an ethanol series (70%, 85% and 100% ethanol for 2 min each) at 4 °C, air-dried and hybridized ON at 37 °C with DNA probes in hybridization buffer (2× sodium saline citrate (SSC), 2 mg/ml BSA, 10% dextran sulfate, 50% deionized formamide) at the final concentration 0.5 pmol/μL. Coverslips were washed as following procedures: 2× SSC/50% formamide, 3 times at 39 °C for 5 mins each; 2× SSC, 3 times at 39 °C for 5 min each; 1× SSC, 5 min at room temperature. DNA oligos probes for TERRA RNA FISH ((TAACCC)_7_-Alexa488-3′) and ((AAT CCC)_7_-Alexa488-3′) were used as a negative control (Integrated DNA Technologies, San Jose, CA, USA). For RNase A treatment, coverslips were incubated with 1 μg/µl RNase A for 1 h at 37 °C prior to hybridization procedures. Nuclei were counterstained with DAPI and slides were mounted with Vectashield (Vector Laboratories, Burlingame, CA, USA). Images were captured at 63× magnification with an Axio Imager.M1 (Carl Zeiss) equipped with a CCD camera and was analyzed with ISIS software (MetaSystems, Milano, Italy). Experiments were repeated at least three times.

### 2.11. Real Time qPCR

Real-time qPCR analysis of TERRA was performed as described [49]. Briefly, RNA was extracted from cells with RNAeasy mini kit (Qiagen, Hilden, Germany) and accurately digested with the RNase-free DNase set (Qiagen). Then, RNA quality was checked on FA gel electrophoresis and amplified in real-time PCR assay with subtelomere-specific primers with a 7900 HT Fast Real-Time PCR System (Applied Biosystem, Waltham, MA, USA).

### 2.12. C-circle Assay

Total DNAs were extracted using Quick C-Circle Lysis Buffer (50 mM KCl, 10 mM Tris HCl pH 8.5, 2 mM MgCl2, 0.5% NP40, 0.5% Tween) and treated with 0.5 μg/μL protease (Qiagen). Samples were placed in thermomixer at 1400 rpm at 56 °C for 1 h than 70 °C for 20 min. Samples, 100 ng in 10-μL each, were combined with 10 μL 0.2 mg/ml BSA, 10% Tween, 100 mM each dATP, dGTP, and dTTP, 1× Φ29 buffer and with or without 7.5 U Φ29 DNA polymerase (NEB) and incubated at 30 °C for 4 h then 70 °C for 20 min. For quantification, the reaction products were dot-blotted onto a 2× SSC-soaked nylon positively charged membrane (GE Healthcare UK Limited, Little Chalfont, UK). DNA was UV–cross-linked onto the membrane, which was then hybridized with a 32P-labeled probe [T3AG2] in Church buffer (0.5 M phosphate buffer pH 7.2, 7% SDS, 1 mM EDTA, 0.1% BSA) overnight at 55 °C. The gel was washed twice and exposed to a PhosphorImager screen and analyzed by Quantity One software (Biorad, Hercules, CA, USA). Signals of samples without Φ29 DNA polymerase were subtracted from signal obtained from corresponding samples with Φ29 DNA polymerase to determine the c-circle expression value.

### 2.13. Western Blot 

Cells were lysed in 20 mM Tris HCl pH 7.5, 150 mM NaCl, 1 mM EDTA, 1% Triton-100X, and protease inhibitors. Protein extracts (15 μg) were loaded on an SDS-PAGE and transferred onto a polyvinylidene fluoride (PVDF) membrane (pore size 0.45 μm; Immobilion-P, Millipore, Massachusetts, MA, USA). Filters were blocked with 3% BSA dissolved in Tris buffered saline (TBS) with 0.05% Tween-20 (TBS-T) for 0.5 h at RT. Membranes were then incubated at 4 °C ON with the following primary antibodies: CHK1 (#sc-8408, Santa Cruz Biotechnology); RAD51 (#sc-33,626; Santa Cruz Biotechnology); Vinculin (#v9131, Sigma-Aldrich). Finally, membranes were incubated 1 h at room temperature with the appropriate HRP-conjugated secondary antibody (Bio-Rad Laboratories, Hercules, CA, USA). Proteins were visualized by the ChemiDoc enhanced chemiluminescence detection system (Bio-Rad, Hercules, CA, USA). Experiments were repeated at least three times. The images were analyzed with Image Lab (Bio-Rad, Hercules, CA, USA).

## 3. Results

### 3.1. RHPS4 Exerts Cytotoxic Effect Over Osteosarcoma Cell Lines Independently From TMM

**Figure 1:**
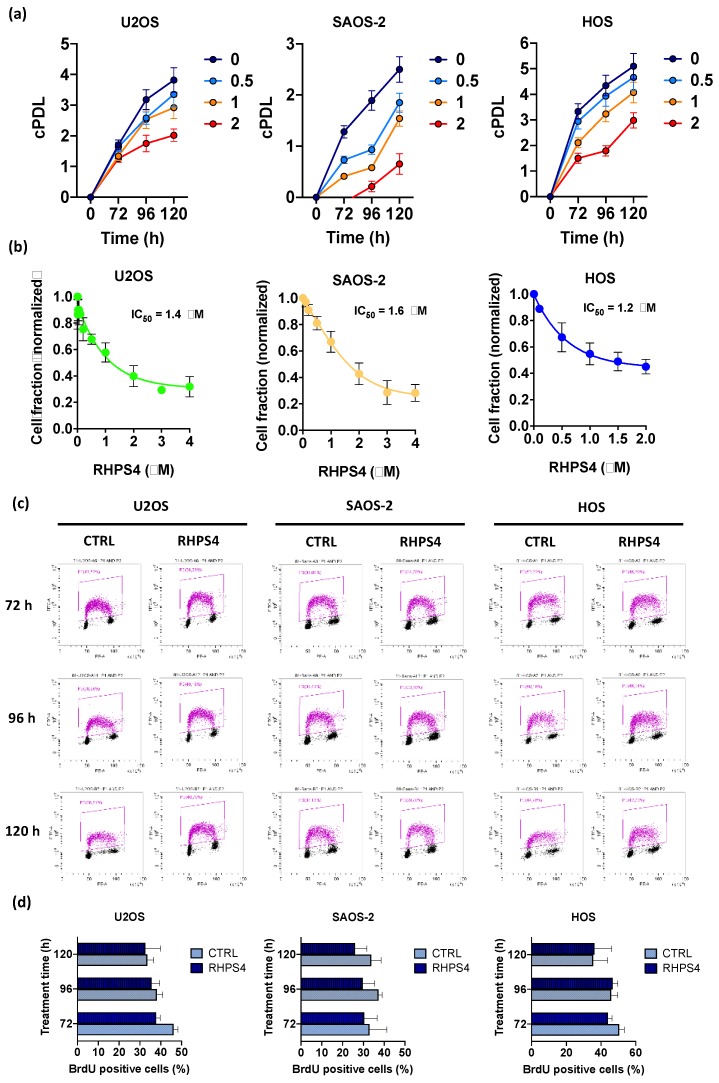
(**a**) Graphs show the cumulative population doubling levels (cPDL) of U2OS, SAOS-2, and HOS. Each cell line was treated with RHPS4 at different concentrations (untreated in blue, 0.5 µM in light blue, 1 µM in yellow, and 2 µM in red) and harvested at different times (72, 96, and 120 h). (**b**) Sensitivity of U2OS, SAOS-2, and HOS osteosarcoma cell lines to RHPS4 concentrations ranging from 0.01 to 4 μM, evaluated after 120 h. These data, obtained by sulforhodamine B (SRB) assay, indicate that RHPS4 sensitivity was comparable in alternative lengthening of telomeres (ALT)-positive (IC50 = 1.4 and 1.6 μM in U2OS and SAOS-2, respectively) and telomerase-positive (IC50 = 1.2 μM in HOS) osteosarcoma cell lines. (**c**) Analysis of the percentage of BrdU incorporating cells as evaluated at 72, 96, and 120 h after RHPS4 treatment. (**d**) Histogram showing the percentage of BrdU incorporating cells excluded S-phase depletion upon RHPS4 treatment at the different fixing times (72, 96, and 120 h). Errors bars denote standard deviations. * *p* < 0.05, ** *p* < 0.01, *** *p* < 0.001 (Student’s *t*-test).

RHPS4 treatment (concentrations ranging from 0.5 to 2 μM) induced a significant and concentration-dependent inhibition of cell growth in both ALT and telomerase positive osteosarcoma cell lines. Growth curves indicated overlapping cell growth inhibition in U2OS, SAOS-2, and HOS independently from their intrinsic doubling times (DT) (DT lower in HOS and U2OS than in SAOS-2; Figure 1a). As shown by IC_50_ values (concentrations of compound leading to 50% inhibition of cell proliferation), calculated from the dose-response curves obtained at 120 h after treatment (Figure 1b), sensitivity to the G4 ligand was comparable in ALT and telomerase positive cell lines (IC_50_ values: 1.6, 1.4, 1.2 μM for SAOS-2, U2OS, and HOS, respectively). 

Progression through cell cycle, and in particular S-phase, was not affected in any of the cell lines analyzed by the treatment with the IC_50_ of the compound. This data suggests that, despite RHPS4 treatment, cells pass through S-phase normally without any significant delay (Figure 1c,d).

### 3.2. RHSP4 Induces Replicative Stress and DNA Damage at Genomic and Telomeric Level in ALT Cells

**Figure 2:**
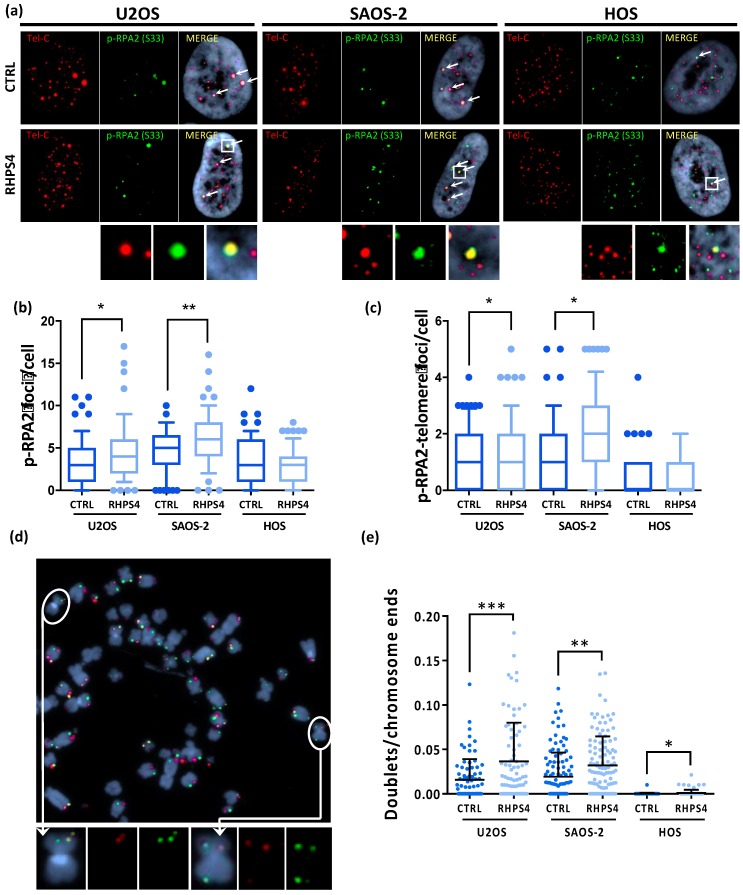
(**a**) Representative images of RHPS4-treated or untreated osteosarcoma cell lines stained for p-RPA2 (S33). For each cell line and condition, single channels (p-RPA2 signals in green and telomeric signals in red) and merged images are illustrated. Arrows indicate p-RPA2 and telomere colocalizations. (**b**) Dotplot showing total amount of p-RPA2 foci per cell. (**c**) Dotplot showing colocalizations between p-RPA2 and telomere. The middle bar denotes median, the box extends from 25th to 75th percentiles and the whiskers above and below the box denote 90th and 10th percentile. (**d**) Representative image of chromosome orientation–FISH (CO–FISH) stained metaphase of SAOS-2 cell line in which double colored doublets are pointed out as shown in enlarged images. (**e**) Dotplot of double color doublets frequency in osteosarcoma cell lines. Black lines denote means and errors bars denote standard deviations. * *p* < 0.05, ** *p* < 0.01, *** *p* < 0.001.

**Figure 3:**
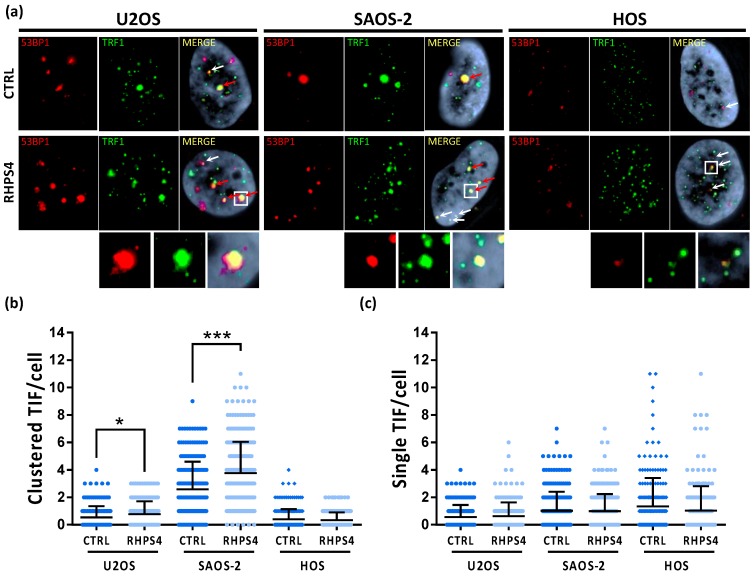
(**a**) Telomere dysfunction induced foci (TIFs) observed by immunofluorescence in osteosarcoma cell lines after 120 h treatment with RHPS4. For each line and condition, single channels (53BP1 protein signals in red and TRF1 protein signals in green) and merged images are illustrated. White arrows indicate TRF1 (green) and 53BP1 (red) colocalizations; red arrows indicate the dysfunctional telomeres aggregation (telomere clusters). (**b**) Dotplot of telomeres clusters formed in ALT cell lines by dysfunctional telomeres aggregation. RHPS4 treatment induces a significant increase in both ALT lines, but not in HOS. (**c**) Dotplot of single colocalizations between TRF1 and 53BP1 showing telomeric localization of DNA damage.. The middle bar denotes mean and the bars above and below the mean denotes standard deviation. * *p* < 0.05, *** *p* < 0.001 (Student’s *t*-test).

Despite the absence of any significant S-phase progression delay, we found that RHPS4 treatment significantly increased replicative stress at both genomic and telomeric levels in ALT cells. Immunofluorescence staining of S33-phospho-RPA2 revealed a significantly higher number of foci in ALT cells treated than in untreated controls (Figure 2a,b). Similarly, replication stress at telomeres, evaluated by the analysis of telomere and S33-phospho-RPA2 signals, was also significantly increased by RHPS4 in ALT cells, whereas any response was observed in HOS cells (Figure 2a,c). Levels of replication stress at telomeres were further analyzed by the scoring of multi-telomeric signals (telomere doublets) after CO–FISH staining. In particular, double color stained doublets were evaluated to score the frequency of replication stress leading to recombinational events at telomeres. As also observed for telomere and S33-phospho-RPA2 colocalizations, we found that telomeric doublet frequency was also higher in U2OS and SAOS-2 than in HOS cells, confirming the evidence of higher basal replication stress at telomeres in ALT cells. In addition, RHPS4 treatment was able to increase very significantly (*p < 0.01*) the frequency of telomeric doublets in ALT cells (2 and 1.5-fold increase in U2OS and SAOS-2, respectively; Figure 2d,e) further confirming the higher sensitivity of ALT cells to G4 ligand-induced replication stress in telomeric regions. To evaluate if replication stress induction was also coupled by an increase in DNA damage at telomeres, the presence of co-localization of the telomere-associated factor TRF1 and the DNA damage marker 53BP1 was evaluated. Unexpectedly, no increase of DNA damage was detected in HOS cells after treatment with the compound neither at genomic nor telomeric level. Conversely, ALT-positive cell lines displayed a significant increase in DNA damage mostly in colocalization with telomeric clusters (Figure 3a,b). In particular, in U2OS cells, the mean of clusterized dysfunctional telomeres increased from 0.5 to 0.8, while in SAOS-2 cells, it increased from 2.6 to 3.8 (Figure 3a,b). No increase in single telomere dysfunction was observed after RHPS4 treatment in either ALT or telomerase positive cells (Figure 3a,c).

### 3.3. RHPS4 Enhances APBs, T-SCE, and C-circles in ALT Cells

**Figure 4:**
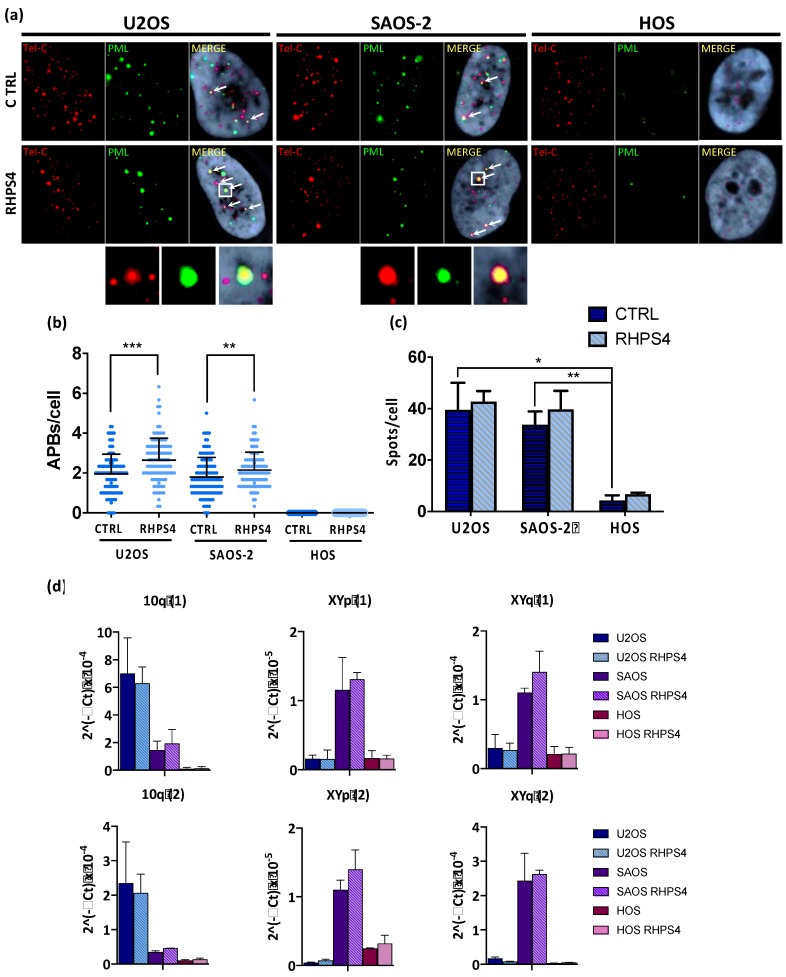
(**a**) Representative images of ALT-associated PML bodies (APBs) in osteosarcoma cell lines after 120 h treatment with RHPS4 obtained by immune-FISH technique. For each line and condition, single channels (PML signals in green and telomeric signals in red) and merged images are illustrated. Arrows indicate PML and telomeres colocalization. (**b**) Dotplot showing colocalizations per cell between telomeres and PML protein. Black lines denote the means and errors bars denote standard deviations. (**c**) Histogram of TERRA frequency in osteosarcoma cell lines obtained by RNA–FISH technique. Data were obtained counting number of spots for each nucleus. Errors bars denote standard deviations. (**d**) qPCR analysis of the expression of TERRA transcribed at different subtelomeres. Errors bars denote standard deviations. * *p* < 0.05, ** *p* < 0.01, *** *p* < 0.001 (Student’s *t*-test).

**Figure 5:**
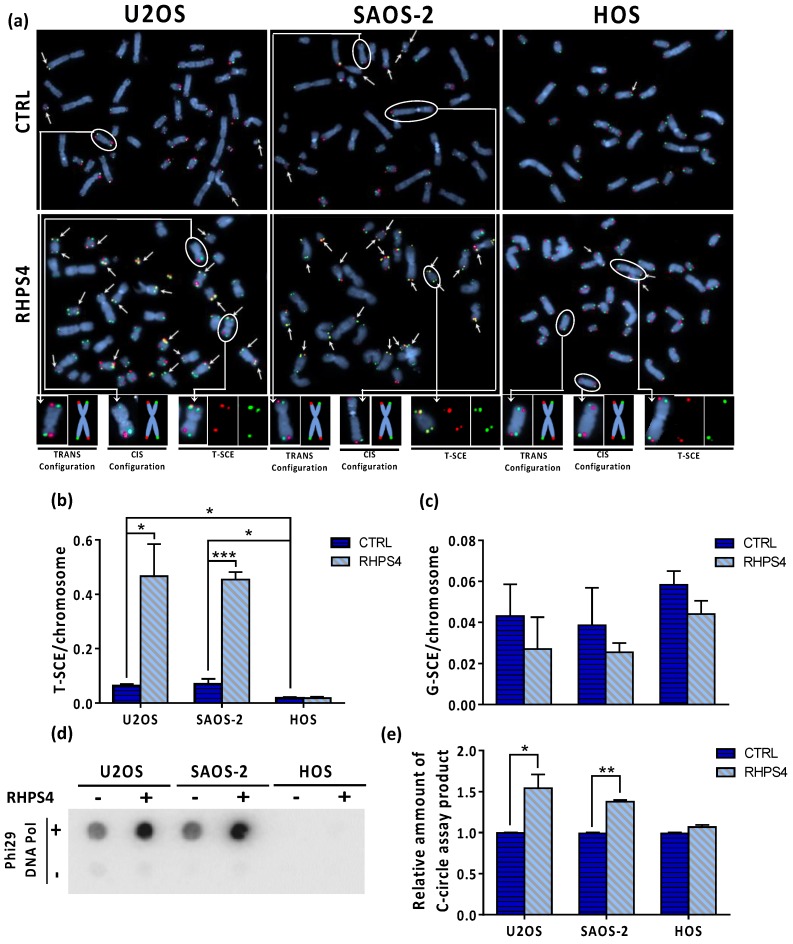
(**a**) Representative images of CO–FISH stained metaphases in U2OS, SAOS-2, and HOS osteosarcoma cell lines after RHPS4 treatment. Arrows indicate telomere sister chromatid exchanges (T-SCE). (**b**) Histograms show T-SCE frequency per chromosome end in osteosarcoma cell lines. RHPS4 treatment induced a significant increase of telomeric sister chromatid exchanges (T-SCE) in U2OS and SAOS-2 but not in HOS cells. (**c**) Genomic sister chromatid exchanges (G-SCE) in U2OS, SAOS-2, and HOS, determined using the method proposed by Cornforth and Eberle [46], are not modulated by RHPS4. (**d**) Dot blot analysis of c-circles in presence or absence of Phi29 DNA Pol enzyme in the indicated cell lines. (**e**) Densitometry of c-circles signals. For each cell line, the background value (-Phi29 DNA Pol sample) was subtracted and reported in histograms. Errors bars denote standard deviations. * *p* < 0.05, ** *p* < 0.01, *** *p* < 0.001 (Student’s *t*-test).

In order to assess whether or not RHPS4 was able to affect the ALT mechanism, the cardinal ALT hallmarks were analyzed. Surprisingly, APBs analysis showed that HOS cells did not display at all PML nuclear bodies. On the contrary, U2OS and SAOS-2 showed a massive presence of this protein at telomeres, as demonstrated by the co-localization of telomeres with PML. Following treatment, the percentage of telomeres colocalizing with PML foci increases in both U2OS and SAOS-2 (1.3-fold increase for both), but not in HOS (Figure 4a,b). In agreement with previously published data [47,50,51], we found that U2OS and SAOS-2 exhibited high levels of TERRA in contrast to telomerase positive HOS cells. However, RHPS4 was not able to alter TERRA levels, as evaluated by RNA FISH and qPCR analysis for subtelomeric regions of chromosomes 10, X, and Y (Figure 4c,d). The strongest evidence supporting the effect of RHPS4 on the ALT pathway comes from the analysis of T-SCE and c-circles. Indeed, RHPS4 determined a significant increase of T-SCE frequency in both ALT cell lines. In particular, U2OS and SAOS-2 showed a 7.3 and a 6.5-fold increase, respectively, whereas no modulation was observed in HOS (Figure 5a,b). Increased recombination rates were not observed at genomic level, as shown by G-SCE analysis (Figure 5a,c). In accordance with T-SCE, a 1.6-fold increase in c-circles amount was found in both U2OS and SAOS-2 upon RHPS4 treatment, whereas the HOS cell line did not show any modulation (Figure 5d,e).

### 3.4. RHPS4 Decreases the Levels of RAD51 and CHK1 Proteins

**Figure 6:**
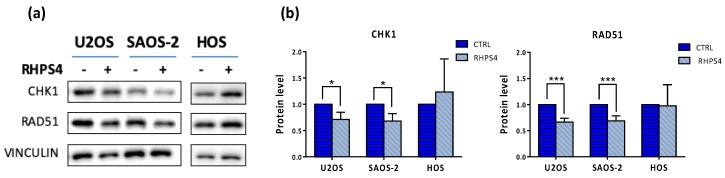
(**a**) Representative image of CHK1 and RAD51 proteins obtained by Western blot analysis. (**b**) Quantitation of protein levels showed a very significant (*p* < 0.001) and a significant (*p* < 0.05) decrease of RAD51 and CHK1 proteins, respectively in both ALT-positive cell lines. Errors bars denote standard deviations. * *p* < 0.05, *** *p* < 0.001 (Student’s *t*-test).

Since CHK1 and RAD51 are well-known players in homologous recombination and were also downregulated upon RHPS4 treatment, as previously reported in glioma cells [33]; we decided to evaluate their levels, in order to gain insight in the recombinational mechanism activated. We noticed a significant 34% and 32% of RAD51 protein decrease in U2OS and SAOS-2, respectively (Figure 6a,b). Similar reduction was also observed for CHK1 (Figure 6a,b). As reported in Figure 1c,d, BrdU incorporating cells quantified through cytofluorimetric analysis exclude that the RAD51 and CHK1 protein reduction upon RHPS4 treatment was due to a depletion of the S-phase.

## 4. Discussion

In the last 20 years, telomeric G4 ligands have been proposed as telomere targeting agents able to rapidly induce telomere dysfunction and growth inhibition in a number of cancer cells both in vitro and in vivo. Interestingly, different G4 ligands (such as quinoline based-ligands, RHPS4, TMPyP4, pyridostatin, BRACO-19, and telomestatin) have been proven to be effective not only in telomerase positive but also in ALT-positive cancer cells [23,24,25,26,52,53]. To find a rationale supporting the observed cell growth inhibitory effect, some authors have raised the possibility that G4 stabilization in telomeric regions might inhibit the ALT-mediated recombination mechanism [35,36,54,55]. Conversely, more recently, other authors reported that G4 stabilizers are able to fuel the ALT mechanisms (both in a RAD51 dependent or independent manner) through the induction of replicative stress and DNA damage at telomeres, in particular in cells harboring ATRX mutations such as ALT cells [37,38]. In the present work, the effect of RHPS4, a well-known and potent telomeric G4 stabilizer, was evaluated in U2OS, SAOS-2 (ALT-positive), and HOS (telomerase positive/ALT-negative) osteosarcoma cell lines, in terms of cell growth inhibition, cell cycle progression, and modulation of the cardinal ALT hallmarks. In agreement with results obtained in ALT positive GM847DM cells [23], RHPS4 was able to reduce cell growth also in U2OS and SAOS-2 osteosarcoma cells (IC50 values: 1.4 and 1.6 μM, respectively). Interestingly, G4 stabilization has been recently proposed as a strategy for the selective targeting of ATRX-deficient gliomas [56]. Indeed, the ATRX protein has been implicated in the direct resolution of G4 secondary structures through its helicase Snf2 domain [57,58] and in the inhibition of RNA–DNA hybrids (R-loops) during transcription that favor G4 formation in the untranscribed DNA strand [59]. In osteosarcoma cells, RHPS4 effectiveness seems to be unlinked from both the genetic status of ATRX and the active TMM, as demonstrated by the very similar sensitivity of HOS telomerase-positive cells to the compound (IC50 value: 1.2 μM). Despite the similar sensitivity, we found that RHPS4 was able to induce genomic and, in particular, telomeric replicative stress preferentially in ALT-positive cell lines. ALT cancer cells exhibit higher basal levels of replication defects, leading to persistent DNA damage at telomeres [37,38]. Abrogation of the G2/M checkpoint, frequently observed in these tumors, may allow cell cycle progression in the presence of incomplete DNA replication, triggering telomere synthesis in the M-phase through the BIR pathway [38]. In particular, we found that S33-p-RPA2/telomere colocalization and telomeric doublets events were significantly increased, indicating that replicative stress in an ALT-permissive genetic background results in telomeric anomalies and possibly DNA damage. Consistent with this observation, analysis of TIFs and APBs confirmed that RHPS4 was able to induce DNA damage in ALT cells, increasing the frequency of DNA damage in clusterized telomeres and PML/telomere colocalizations. Ultrabright telomeric signals are a well-known marker of ALT and it is widely accepted that, in ALT cells, damaged telomeres converge in nuclear subcompartments together with PML protein and other proteins that mediate homologous recombination (HR) and/or break-induced repair (BIR) [60]. The increase of telomeric DNA damage and APBs was not coupled by an increase of TERRA expression, as shown by RNA–FISH and qPCR experiments, but was strongly supported by T-SCEs analysis. Data showed that T-SCEs (but not G-SCEs) greatly increased after RHPS4 treatment in both U2OS and SAOS-2 cells, whereas no changes were observed in HOS cells as well as in telomerase positive glioblastoma U251MG cells (data not shown). To further corroborate these data c-circles analysis, we also considered the most reliable ALT marker for diagnostic purposes [61], which were significantly induced in ALT-positive cells but not in the HOS cell line. Taken together, ALT hallmark analysis indicates that telomeric replicative stress induced by the G4 ligand RHPS4 is able to increase DNA damage at telomeres fueling the ALT pathway in ALT-positive osteosarcoma cells. However, our data do not clarify which kind of ALT pathway is activated in response to RHPS4. Very recently, we showed that RHPS4 is able to strongly downregulate CHK1 and RAD51 proteins in glioblastoma cancer cells, probably due to specific targeting of G4 located in promoters of these genes [33]. Interestingly, RAD51 is implicated in HR-mediated telomere lengthening in ALT cells but not in BIR [62]. Protein level analysis showed a 30–35% reduction of RAD51 in both ALT-positive cell lines, suggesting that HR may be partially inhibited and pointing to a major role of BIR in response to RHPS4. Recently the group of Jerry Shay demonstrated that pyridostatin treatment is able to induce BIR in ALT cells, and our data seem to go in the same direction [38]. However, which kind of ALT pathway is activated in response to RHPS4 is still unclear to us, and further experiments are needed to clarify this aspect. 

## 5. Conclusions

In conclusion, the present work indicates that, despite its efficacy to inhibit cell growth, the G4-ligand RHPS4 is able to stimulate telomeric recombination and c-circles formation through the induction of telomeric replicative stress, telomeric DNA damage, and APBs formation in U2OS and SAOS-2 ALT osteosarcoma cells. These are in accordance with with the emerging role of G4 secondary structures and telomeric replicative stress as triggers of the recombination-mediated telomere lengthening in an ALT permissive (ATRX-deficient) genetic background [37,38]. ALT fueling by G4 stabilizing compounds raises questions on their possible employement as therapeutic options to target ALT-positive tumors, as recently proposed for gliomas [56].
